# Optimizing Differentiated HIV Treatment Models in Urban Zimbabwe: Assessing Patient Preferences Using a Discrete Choice Experiment

**DOI:** 10.1007/s10461-020-02994-z

**Published:** 2020-08-18

**Authors:** Michael Strauss, Gavin George, Joanne E. Mantell, Munyaradzi Mapingure, Tsitsi B. Masvawure, Matthew R. Lamb, Jennifer M. Zech, Godfrey Musuka, Innocent Chingombe, Martin Msukwa, Rodrigo Boccanera, Clorata Gwanzura, Tsitsi Apollo, Miriam Rabkin

**Affiliations:** 1 Health Economics and HIV/AIDS Research Division (HEARD), University of KwaZulu Natal, Durban, South Africa; 2 Department of Psychiatry, Division of Gender, Sexuality and Health, The New York State Psychiatric Institute and Columbia University Irving Medical Center, New York, NY USA; 3 ICAP at Columbia University, Harare, Zimbabwe; 4 Health Studies Program, Center for Interdisciplinary Studies, College of the Holy Cross, Worcester, MA USA; 5 ICAP at Columbia University, New York, NY USA; 6 Department of Epidemiology, Columbia University Mailman School of Public Health, New York, NY USA; 7 ICAP at Columbia University, Pretoria, South Africa; 8 Health Resources and Services Administration (HRSA), Rockville, MD USA; 9 HIV/AIDS and STIs Unit, Ministry of Health and Child Care, Harare, Zimbabwe

**Keywords:** HIV treatment, Discrete choice experiment, Differentiated service delivery, Zimbabwe, Urban, Tratamiento del VIH, experimento de elecciones discretas, prestación de servicios diferenciados, Zimbabue, urbano

## Abstract

Differentiated service delivery holds great promise for streamlining the delivery of health services for HIV. This study used a discrete choice experiment to assess preferences for differentiated HIV treatment delivery model characteristics among 500 virally suppressed adults on antiretroviral therapy in Harare, Zimbabwe. Treatment model characteristics included location, consultation type, healthcare worker cadre, operation times, visit frequency and duration, and cost. A mixed effects logit model was used for parameter estimates to identify potential preference heterogeneity among participants, and interaction effects were estimated for sex and age as potential sources of divergence in preferences. Results indicated that participants preferred health facility-based services, less frequent visits, individual consultations, shorter waiting times, lower cost and, delivered by respectful and understanding healthcare workers. Some preference heterogeneity was found, particularly for location of service delivery and group vs. individual models; however, this was not fully explained by sex and age characteristics of participants. In urban areas, facility-based models, such as the Fast Track model requiring less frequent clinic visits, are likely to better align with patient preferences than some of the other community-based or group models that have been implemented. As Zimbabwe scales up differentiated treatment models for stable patients, a clear understanding of patient preferences can help in designing services that will ensure optimal utilization and improve the efficiency of service delivery.

**Electronic supplementary material:**

The online version of this article (10.1007/s10461-020-02994-z) contains supplementary material, which is available to authorized users.

## Resumen

La entrega de servicios diferenciados representa una gran promesa para optimizar la prestación de servicios sanitarios para el VIH. Este estudio utilizó un experimento de elecciones discretas para evaluar las preferencias en cuanto a las características de los modelos diferenciados de prestación de tratamiento para el VIH entre 500 adultos con supresión viral bajo tratamiento antirretroviral en Harare, Zimbabue. Las características de los modelos de tratamientos incluyeron el lugar, el tipo de consulta, la estructura en la que estaba inserto el trabajador sanitario, los tiempos operativos, la frecuencia y duración de las visitas y los costos. Se utilizó un modelo de probabilidad de efectos mixtos para las estimaciones de los parámetros para identificar una posible heterogeneidad en las preferencias entre los participantes, y se estimaron los efectos de las interacciones por el sexo y la edad como fuentes potenciales de diferencia en las preferencias. Los resultados indicaron que los participantes preferían servicios basados en centros de salud, visitas menos frecuentes, consultas individuales, tiempos de espera más cortos y menores costos, proporcionados por trabajadores sanitarios respetuosos y comprensivos. Se hallaron algunas heterogeneidades en las preferencias, particularmente para el lugar de la prestación del servicio y para los modelos grupales frente a individuales; sin embargo, esto no se explicó totalmente por las características de sexo y edad de los participantes. En áreas urbanas, los modelos basados en los centros, como el modelo de atención rápida (Fast Track), que requieren visitas menos frecuentes a la clínica, probablemente concuerden mejor con las preferencias de los pacientes que los otros modelos grupales o comunitarios que se han propuesto. A medida que en Zimbabue se amplíen los modelos de tratamiento diferenciado para los pacientes estables, un claro conocimiento de las preferencias de los pacientes puede ayudar a diseñar servicios que garantizarán una utilización óptima y mejorarán la eficiencia de la prestación de servicios.

## Introduction

Zimbabwe’s Ministry of Health and Child Care (MoHCC) has made significant progress in scaling up HIV services. UNAIDS estimates that 88% of Zimbabwe’s 1.3 million people living with HIV were on antiretroviral therapy (ART) in 2018 and that the country has seen a 38% drop in new HIV infections and a 60% drop in AIDS-related deaths since 2010 [1]. In order to sustain these achievements, the MoHCC and its partners plan to expand access to ART while ensuring existing patients are retained in care and adhere to treatment. In the context of Zimbabwe’s limited resources and strained health system, the MoHCC launched a differentiated service delivery (DSD) strategy for HIV, in which stable adult patients on ART were moved to less-intensive treatment models [2]. Stable patients are defined as having been on ART for at least six months, doing well on treatment (e.g., having a viral load of < 1000 copies/mm^3^ or a CD4 count of > 200 cells/mm^3^) and lacking co-morbidities or psychosocial contraindications to less-intensive care [2].

Zimbabwe has been an early adopter of differentiated ART (DART), which is a global strategy endorsed by the World Health Organization (WHO) and implemented by countries around the world [3, 4]. DART moves away from a “one size fits all” to a patient-centered approach, enabling stable patients on ART to opt into models with fewer and faster visits to health facilities or community-based services [5]. By putting patients at the center of ART delivery and tailoring variables such as visit frequency, visit location and health care worker (HCW) cadre, DART can enhance the quality and efficiency of health services, as well as improve both patient satisfaction and alleviate the burden on the health system [6, 7].

When Zimbabwe initiated DART services in 2017, five DART models were included in its national guidelines [2]. These included community-based ART refill groups (CARGs), facility-based group refill club, fast track appointments, family ART pickups and an outreach model in which health workers bring treatment services to locations in the community. Table 1 highlights the key characteristics of these DART models.

**Table 1:** Characteristics of DART models in Zimbabwe

	Fast track	Club refill	Outreach	Community ART refill groups (CARGs)	Family member refill
Location	Clinic-based	Clinic-based	Community-based	Community-based (except for the person who collects drugs for the group at a facility)	Clinic-based (for the member of the family who collects medication)
Time	30 min	Depends on the group, but should be between 45 and 75 min	Appointments are scheduled—time will depend on efficiency at the point of care	Depends on the group, but does not include consultation with a healthcare worker	Depends on the model used at the clinic
Provider	Healthcare worker	Healthcare worker-led	Healthcare worker/expert patient	Peer-led	Family member
Participants	Individual	Group	Individual	Group	Group
Times of operation	Clinic hours	Clinic hours	Flexible	Flexible	Clinic hours
Visit spacing	3 months	3 months	3 months	6 months	Varied

*Source* Adapted from Zimbabwe Ministry of Health and Child Care Operational and Service Delivery Manual for the Prevention, Care and Treatment of HIV in Zimbabwe, 2017

In order for DART to achieve the goals of increasing the quality and efficiency of HIV treatment services, it requires implementation at scale. In November 2018, the MoHCC estimated that 35% of people on ART had been shifted to DART models and set a goal of 65% coverage by December 2019 and 80% coverage by December 2020 [8]. Early programmatic data suggest that uptake of community-based models is highest in rural areas and that uptake of facility-based models is higher in urban areas, but it is not clear whether these enrollment patterns are due to supply or demand. Additional information about patient preferences will help identify which DART models should be prioritized and in which settings. In response, ICAP at Columbia University partnered with the MoHCC and the U.S. Health Resources and Services Agency (HRSA) to conduct a discrete choice experiment (DCE) to explore the preferences of stable patients on ART in Harare, Zimbabwe.

This study builds on previously published work [9] by exploring in greater detail the heterogeneity in preferences of participants, to better understand how preferences might diverge depending on the age and sex of patients, given the differences in how these characteristics might interact as individuals access ART services. This work examined commonalities that emerged among findings from qualitative focus groups and in-depth interviews, a quantitative survey and some of the early DCE analyses [9]. This paper deepens the analysis into preference heterogeneity in the choices made by participants regarding the characteristics of different DART models in Harare, Zimbabwe, in two ways: (1) using more sophisticated statistical modeling techniques that allow for preference heterogeneity, and (2) exploring divergence in preferences by age and sex more in depth to understand whether tailored DART models focused on sex or age might be appropriate for increasing demand.

## Methods

The DCE is a quantitative technique embedded in well-established economic theory, used to elicit information about preferences and key drivers of choice, by offering participants hypothetical scenarios (“choice sets”) that force trade-offs between the key characteristics of goods or services [10–12]. This methodology has been used to inform health policy and is endorsed by the WHO as a useful tool for health research in low-income settings [13–16]. DCEs enable the valuation of individual attributes (e.g., cost, service quality) that comprise a scenario (e.g. health service package, like a DART model) and thus can be used to determine the specific characteristics of services of highest value to the target population.

### Study Setting

Seven of the 51 public health facilities that deliver ART in Harare, Zimbabwe, were purposively sampled for inclusion in the study, selected in partnership with the MoHCC and a study advisory group. Selection criteria included location in Harare, high-volume ART sites, implementation of DART and geographical representation of Harare medical facilities shown in Table 2.

**Table 2:** DCE site characteristics in Harare, Zimbabwe

Facility name	District	Adults on ART^a^	DART initiated^b^	Types of DART models	Number of participants recruited (% in brackets)
St. Mary’s Clinic	Chitungwiza Urban District	6409	< 10%	CARGs Fast track Family member refill Club refill	74 (14.8%)
Mabvuku PolyClinic	Eastern District	4097	< 10%	CARGs Fast track	73 (14.6%)
Wilkins Infectious Disease Hospital	Central Harare	2616	Data not available	Fast track Family member refill	73 (14.6%)
Rujeko PolyClinic	North Western District	6985	60–80%	CARGs Fast track Family member refill	70 (14.0%)
Highfield PolyClinic	South Western District	4757	60–80%	CARGs	71 (14.2%)
Rutsanana PolyClinic	South Western District	3984	< 10%	CARGs Fast track	66 (13.2%)
Budriro PolyClinic	West South West District	6020	30–50%	CARGs Fast track Family member refill	73 (14.6%)

^a^*Source* MoHCC Zimbabwe programme data, ART Summary, June 2019

^b^DSD Quarterly Report, Q2 2019

### Study Design

In preparation for the DCE, an initial phase of qualitative work was conducted in July 2018 to identify DART characteristics or attributes to include in the DCE, and to ensure that the language and illustrations used in the choice sets presented to participants were contextually appropriate and understandable. To this end, we conducted 35 in-depth interviews (IDIs) with healthcare providers and eight focus group discussions (FGDs) with patients on ART (N = 54) to explore HIV treatment and DSD model preferences. At the end of each FGD, participants engaged in a ranking exercise of all of the attributes that emerged from the FGDs. Attributes were listed on a board and each participant was given 20 stickers (a different color for each participant) and asked to place them on the board next to the attributes s/he felt were most important. Stickers were tallied across all FGDs and the most highly ranked attributes, as well as appropriate levels (possible alternatives/options for each attribute), were selected for inclusion alongside the attributes and levels that specifically relate to the characteristics of the different DART models currently being used in Zimbabwe.

Following selection of attributes and levels, we conducted two additional FGDs with patients on ART (N = 16) to obtain feedback on the potential images/illustrations of these attributes. Table 3 shows the final list of attributes and levels, along with definitions used in this study.

**Table 3:** Final attributes, levels and definitions included in the DCE design

Attribute		Level with definition
Location of service delivery	1	Health facility/clinic close to home or workplace (10 min travel)^a^: A health facility (e.g., clinic or hospital), which is 10 min in travel time from your home or workplace
	2	Health facility/clinic further from home or workplace (45 min travel): A health facility (e.g., clinic or hospital), which is 45 min in travel time from your home or workplace
	3	Community-based DART services: In the community (e.g., at a central point, like a school, community hall, church, etc.)
	4	Home-based DART services: At home
Participants/others seen at same visit	1	Individual^a^: By yourself
	2	Group: With a group of other ART patients and/or family members
Type of service provider The person/people who leads delivery of services (counseling, weight and vital signs, symptoms screening, adherence assessment and/or ART distribution)	1	Professional health worker who is respectful and understanding^a^: A professional health worker (e.g., nurse or doctor) who is respectful and understanding
	2	Professional health worker who is *not* respectful and understanding: A professional health worker (e.g., nurse or doctor) who is *not* respectful and understanding
	3	Peer/lay person who is respectful and understanding: A peer (someone on ART) or lay person (e.g., a lay counselor or community health worker) who is respectful and understanding
	4	Peer/lay person who is *not* respectful and understanding: A peer (someone on ART) or lay person (e.g., a lay counselor or community health worker) who is *not* respectful and understanding
Times (days and hours) of operation Days and times ART services are provided	1	Work week only (standard hours: 8am–4pm)^a^: Monday through Friday from 8am to 4pm
	2	Work week with early morning hours (opens at 5am): Monday through Friday from 5am to 4pm
	3	Work week with evening hours (open until 8 pm): Monday through Friday from 8am to 8pm
	4	Work week + weekend hours (7 days a week, 8am–4pm): 7 days a week from 8am to 4pm
Frequency of visits/visit spacing Frequency of routine visits for ART refill	1	Four times a year (every three months)^a^: Four times a year (or every three months)
	2	Two times a year (every six months): Two times a year (or every six months)
Total time for visit Including registration, wait times, and time with providers. Does not include transportation time	1	30 min: The total time you spend (including waiting time and time with providers) for your visit is 30 min. This does not include travel time
	2	1 h^a^: The total time you spend (including waiting time and time with providers) for your visit is 1 h (60 min). This does not include travel time
	3	2 h: The total time you spend (including waiting time and time with providers) for your visit is 2 h (120 min). This does not include travel time
	4	4 h: The total time you spend (including waiting time and time with providers) for your visit is 4 h (240 min). This does not include travel time
Total cost of visit Including transportation, direct medical costs (e.g., consultation or booking fee, lab costs if not available at public facility, non-ARV drug costs), costs of childcare	1	Free: The total cost of your visit is free
	2	$1^a^: The total cost of your visit is $1. This includes transportation, health services (e.g., consultation or booking fee, lab costs, non-ARV drug costs) and childcare
	3	$3: The total cost of your visit is $3. This includes transportation, health services (e.g., consultation or booking fee, lab costs, non-ARV drug costs) and childcare
	4	$10: The total cost of your visit is $10. This includes transportation, health services (e.g., consultation or booking fee, lab costs, non-ARV drug costs) and childcare

^a^Levels labeled with an asterisk were used as reference levels in the statistical analysis

### DCE Instrument Design

Given the large number of attributes and levels, we used a fractional factorial, unlabeled design with binary choice sets where individuals were offered the option between two hypothetical DART models, with no opt-out option, to maximize the amount of information about participants’ willingness to make trade-offs between attributes, even for individuals who have very low levels of acceptability of DART [3]. Following the method set out in Street et al. [17], an orthogonal main effects plan (OMEP) of 32 profiles (combinations of different levels for each attribute) was generated using SPSS 23.0; these profiles were used as the first alternative in each choice set. To generate the second alternative for every choice, one level was added (cyclically) to each level in the first alternative. This approach yields an optimal design using the D-efficiency criterion, and also adheres to the design principles set out in Zwerina et al*.* [18]—minimum overlap, orthogonality, level and utility balance. The 32 choice sets were divided into four versions each with eight choice sets by including a blocking variable into the OMEP to retain design efficiency; participants were randomly assigned to one version.

### Sample Size

The optimal number of participants needed for statistical power is determined by the number of choices in each choice set, the number of attributes, and the number of levels for each of the attributes, as well as the true value of unknown parameters that are to be estimated in the discrete choice models. The rule of thumb often used to calculate a useful minimum sample size [19], N, is:

$$N \ge 500\frac{L}{SJ}$$

where L is the maximum number of levels for any attribute, S is the number of choices in each choice set, and J is the number of tasks (or choices) presented to each participant. In the case of this DCE, the highest number of levels in any attribute is four, and participants are presented with eight binary choices each (from the total design of 32). Thus, we recruited a minimum sample size of 125 participants per subgroup (younger men/younger women/older men/older women) for a total sample size of 500. We used age categories 29 years and younger; and 30 years and older, given the typical age distribution of stable ART patients at the facilities included in the study.

### Eligibility Criteria

Participants were eligible for inclusion in the DCE if they: (1) were 18 years and older; (2) were HIV-positive and on ART; (3) met “stable” criteria as per national guidelines; (4) were not currently participating in a DART model; (5) had lived in Harare for at least one year; and (6) spoke English and/or Shona. The rationale for enrolling participants who were not currently enrolled in DART was that we were most interested in understanding the characteristics that could potentially be barriers for new DART patients, and characteristics that could increase overall utility for patients who have yet to enroll.

### DCE Implementation

**Fig. 1:**
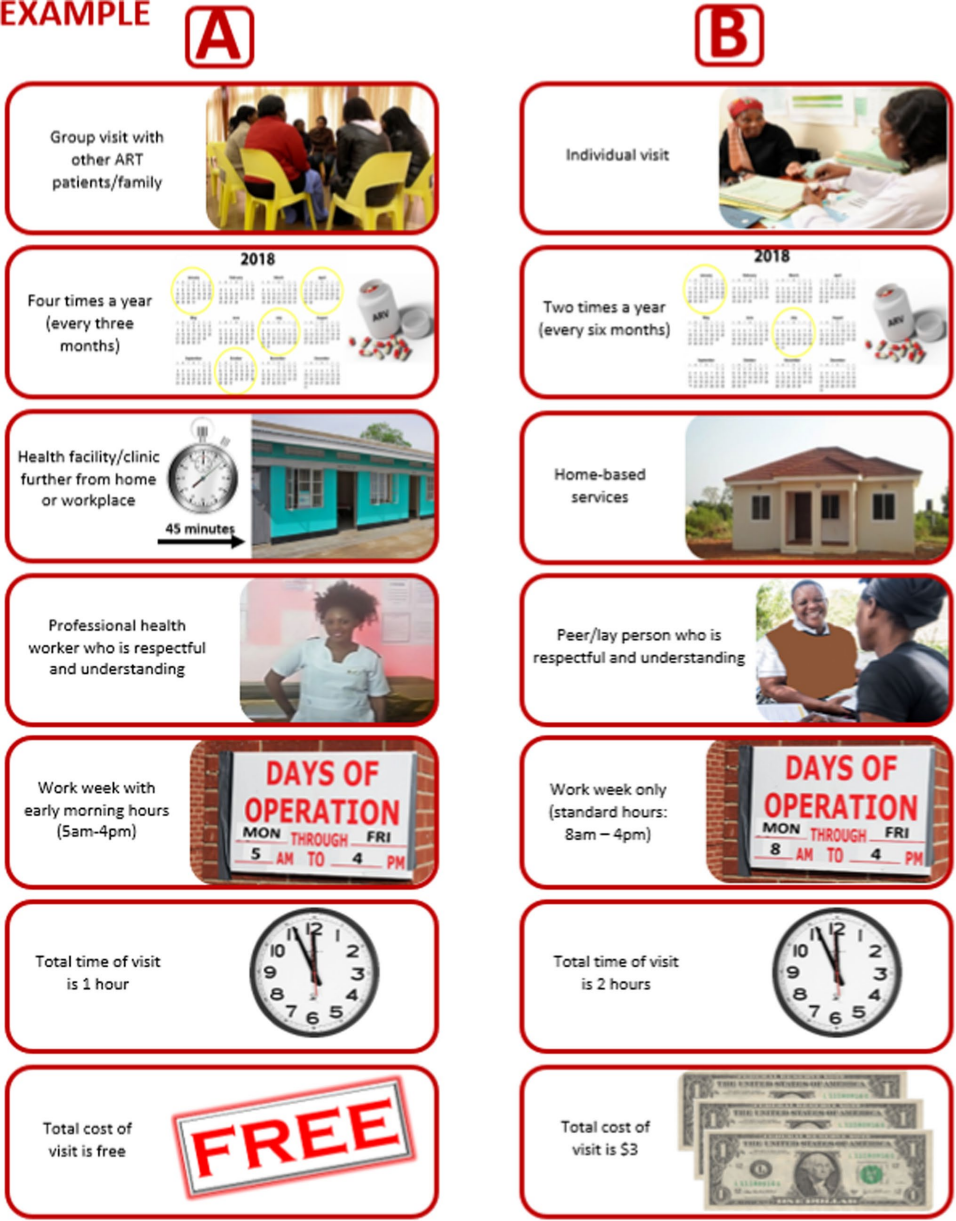
Example of a choice set

The DCE was conducted between October and November 2018, as part of a larger quantitative survey. Trained study staff explained the DCE and obtained informed consent from participants. Each participant was then given nine or ten choices (eight from the design with a repeat question to check for consistency of responses) between two hypothetical DART models. Choice sets were illustrated on “choice cards”, which were laminated and bound together in a booklet, with each choice set shown using pictures and labels in English (Fig. 1 shows an example of a single choice set). Participant choices were captured on electronic tablets.

### Statistical Analysis

We used a mixed effects logit model allowing random effects for all parameters and using Halton draws with 1000 replications to estimate the relative utility for each of the attributes and levels, and using dummy variable coding for each attribute level. Mixed effects models allow for relaxing the assumption of the independence of irrelevant alternatives (IIA) and an assessment of heterogeneity in preferences across attributes [10]. Fixed and random effects logistic regression models were also run for comparison purposes along with a Hausman specification test [20], which revealed no evidence of violations in the IIA assumption, suggesting a fixed effects model is more appropriate than a random effects logit model for estimation. The main effects mixed logit, fixed effects logit and random effects logit models produced similar results in terms of the magnitude and direction of odds ratios and significance of preferences for each attribute level (see electronic supplementary material). Fixed and random effects logit models are often used for estimating model parameters in DCEs that employ a binary design [15, 21]. These models estimate the probability of choosing one alternative over another, such that:

$$Pr_{ij} = \frac{{{\exp}\left( {\beta X_{ij} } \right)}}{{\mathop \sum \nolimits_{k = 1}^{K} {\exp}\left( {\beta X_{ik} } \right)}},\,{\text{for}}\,{\text{ both}}\,{\text{ alternatives}}\,{\text{ J}}\,{\text{ in}}\,{\text{ the}}\,{\text{ choice}}\,{\text{ set}},$$

where Prij is the probability of individual i choosing alternative j from a set of alternatives k, β is a column vector of parameter estimates associated with Xij, which is a row vector of the levels of the attributes in alternative j chosen by individual i [22]. The mixed effects logit model allows for preference heterogeneity between participants by allowing parameter estimates to vary over individuals in the population with density $f(\beta |\theta )$, assumed to be standard normal in this analysis [12]. The parameter estimates can thus be obtained by calculating the probabilities from the standard logit formulation over all possible values of β i.e. the unconditional choice probability that individual i chooses alternative j for choice set t becomes the integral of the logit specification over all values of β such that:

$$Pr_{ijt} \left( \theta \right) = \int {\frac{{\exp \left( {\beta X_{ijt} } \right)}}{{\mathop \sum \nolimits_{k = 1}^{K} \exp \left( {\beta X_{ikt} } \right)}}f\left( {\beta {|}\theta } \right)d\beta }.$$

The main effects only, mixed effects logistic regression model is presented in Model 1. The results of the fixed effects logit model previously published are included in the electronic supplementary material for reference [9].

To understand if preferences differ by age and sex, two types of sub-analysis were conducted. First, we generated dummy variables for sex, age and for age and sex combined^1^ (younger men; younger women; older men; older women). In order to run interaction models, each of these dummy variables was multiplied by the dummy variables for each attribute level, and these new variables were included along with the original dummy variables in the fixed effects logit models. Model 2 was an interaction model comparing the preference structures of men to those of women; Model 3 was an interaction model comparing the preference structures of older participants to those of younger participants. In order to see how the preferences of particular groups of interest diverged from the overall preferences of the sample (men age 18–29; women age 18–29; men age 30 and older; women age 30 and older), we ran four separate interaction models (Models 4–7). This analysis highlights specific attributes and levels where preferences diverge for specific groups. To compare preferences across all the groups, the second component of the sub-analysis used main effects only fixed effects logit models, stratified by sex (Model 8), age (Model 9), and age by sex (Model 10–13). Models 2–7 illustrated *differences* in preference structures between groups, while Models 8–13 were important for understanding overall preference structures within each stratification. Finally, to better understand how preferences regarding the type of provider were driven by the type of HCW (i.e. professional HCW vs. peer/lay counselor) and the HCW attitudes (respectful and understanding vs. *not* respectful or understanding), we created separate dummy variables for these two characteristics and analyzed these preferences using a fixed effects logit model for main effects only (Model 14). All analyses were conducted in Stata 15 [23]. The results of the analysis are presented as odds ratios in relation to a baseline scenario which included the reference levels for each attribute (see Table 2). The results of the analyses that are not presented in the text are included in the electronic supplementary material.

### Ethical Reviews

The protocol was approved by the Columbia University Institutional Review Board (Protocol IRB-AAAR9020), the Medical Research Council of Zimbabwe (Protocol MRCZ/A/2326), and the U.S Health Resources & Services Administration (HRSA). Written informed consent was obtained from all participants.

## Results

### Characteristics of the Sample

As per the sampling strategy, 125 younger men, 125 younger women, 125 older men and 125 older women were included in the study, using age categories of 29 years and younger *vs*. 30 years and older. The median age of the sample was 29.5 years (interquartile range was 24 to 41 years old). Participant demographics and health status are described in Table 4.

**Table 4:** DCE participant demographics

	Total sample	Men (n = 250)	Women (n = 250)
	N (%)	n (%)	n (%)
Age			
18–29 years	250 (50.0%)	125 (50.0%)	125 (50.0%)
30 years and older	250 (50.0%)	125 (50.0%)	125 (50.0%)
Educational attainment			
None	1 (0.2%)	0 (0.0%)	1 (0.4%)
Primary	55 (11.0%)	28We (11.2%)	27 (10.8%)
Secondary	401 (80.2%)	192 (76.8%)	209 (83.6%)
> Secondary	43 (8.6%)	30 (12.0%)	13 (5.2%)
Marital status			
Single	143 (28.6%)	90 (36.0%)	53 (21.2%)
Married monogamous	248 (49.6%)	119 (47.6%)	129 (51.6%)
Married polygamous	9 (1.8%)	1 (0.4%)	8 (3.2%)
Living with partner	8 (1.6%)	3 (1.2%)	5 (2.0%)
Divorced/separated	52 (10.4%)	28 (11.2%)	24 (9.6%)
Widowed	40 (8.0%)	9 (3.6%)	31 (12.4%)
Spouse/partner HIV status among those married or living with partner (n = 265)			
Positive	191 (72.0%)	88 (71.5%)	103 (72.5%)
Negative	61 (23.0%)	33 (26.8%)	28 (19.7%)
Don't know/refused/missing	13 (4.9%)	2 (1.6%)	11 (7.7%)
Children under 18 in household			
Yes	337 (67.4%)	154 (61.6%)	183 (73.2%)
No	163 (32.6%)	96 (38.4%)	67 (26.8%)
HIV-positive family member			
Yes	339 (67.8%)	162 (64.8%)	177 (70.8%)
No	137 (27.4%)	75 (30.0%)	62 (24.8%)
Don't know	24 (4.8%)	13 (5.2%)	11 (4.4%)
Currently working			
Yes	256 (51.2%)	159 (63.6%)	97 (38.8%)
No	244 (48.8%)	91 (36.4%)	153 (61.2%)
Primary occupation among those currently working (n = 256)			
Professional	65 (25.4%)	48 (30.2%)	17 (17.5%)
Self-owned business	89 (34.8%)	44 (27.7%)	45 (46.4%)
Other business	30 (11.7%)	18 (11.3%)	12 (12.4%)
Services	50 (19.5%)	33 (20.8%)	17 (17.5%)
Sex worker	1 (0.4%)	0 (0.0%)	1 (1.0%)
Other	21 (8.2%)	16 (10.1%)	5 (5.2%)
Income last month among those currently working (n = 256)			
≤ $100	87 (34.0%)	41 (25.8%)	46 (47.4%)
$101–500	120 (46.9%)	85 (53.5%)	35 (36.1%)
> $500	44 (17.2%)	31 (19.5%)	13 (13.4%)
Don't know/refused/missing	5 (2.0%)	2 (1.3%)	3 (3.1%)
Bothered by physical symptoms related to HIV infection			
Not at all	279 (55.8%)	150 (60.0%)	129 (51.6%)
A little	98 (19.6%)	44 (17.6%)	54 (21.6%)
A moderate amount	76 (15.2%)	44 (17.6%)	32 (12.8%)
Very much	24 (4.8%)	7 (2.8%)	17 (6.8%)
An extreme amount	22 (4.4%)	5 (2.0%)	17 (6.8%)
Don’t know	1 (0.2%)	0 (0.0%)	1 (0.4%)
Amount of money paid at the health facility at most recent visit			
Nothing	94 (18.8%)	52 (20.8%)	42 (16.8%)
< $1	9 (1.8%)	5 (2.0%)	4 (1.6%)
$1–3	395 (79.0%)	192 (76.8%)	203 (81.2%)
> $3	2 (0.4%)	1 (0.4%)	1 (0.4%)
Amount of time spent at the health facility at most recent visit			
Less than 30 min	60 (12.0%)	28 (11.2%)	32 (12.8%)
30 min–1 h	128 (25.6%)	51 (20.4%)	77 (30.8%)
Between 1–2 h	138 (27.6%)	78 (31.2%)	60 (24.0%)
Between 2–4 h	121 (24.2%)	67 (26.8%)	54 (21.6%)
> 4 h	50 (10.0%)	25 (10.0%)	25 (10.0%)
Don’t know/refused/missing	3 (0.6%)	1 (0.4%)	2 (0.8%)

### Main Effects

**Fig. 2:**
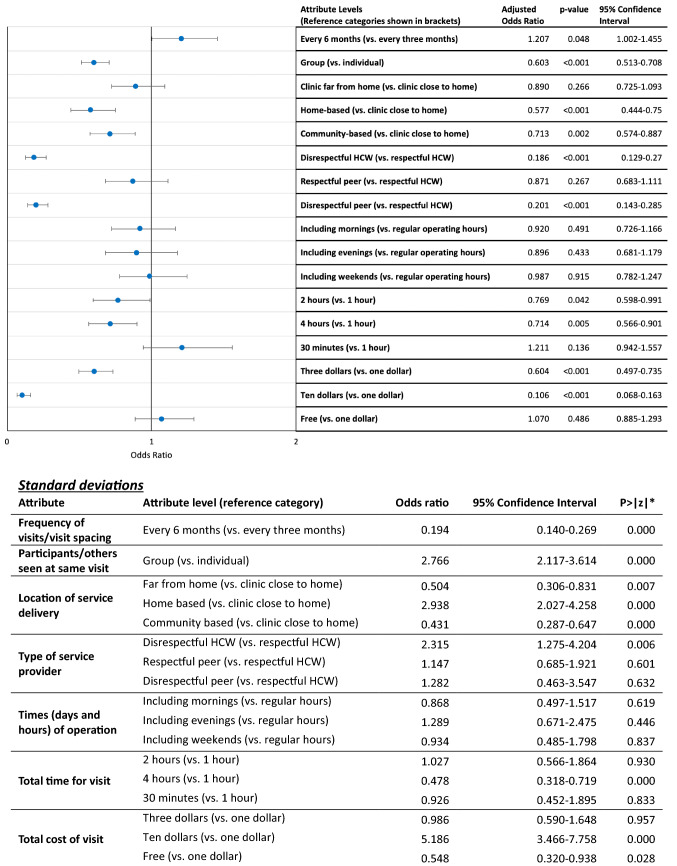
Main effects only mixed effects logistic regression means (Model 1)

Figure 2 shows the results of the main effects mixed effects logit analysis, which included all 500 participants. Overall, there was a significant preference for a six-monthly medication collection schedule rather than a three-monthly collection, although the overall effect on preferences was found to be relatively small (OR 1.207; 95% CI 1.002–1.455). Participants did not prefer to collect their medication from a community-based collection point compared to a clinic close to home (OR 0.713; 95% CI 0.574–0.887), nor did they prefer a home-based (door-to-door) delivery model compared to a clinic close to home (OR 0.577; 95% CI 0.444–0.750). In terms of the location of the service, participants’ preferences were not significantly different between services at a clinic close to home (about 10 min travel time) and a clinic far from their home (travel time 45 min). Our findings indicate that participants had a relatively strong preference against group ART delivery compared to individual ART delivery (OR 0.603; 95% CI 0.513–0.708). Alternative operating hours of the delivery models had no significant effect on preference structures.

The results from Model 1 suggest that there was no significant difference in preferences between ART delivery models that were delivered by a HCW or a peer/lay counselor, provided the person was respectful and understanding. This was one of the most important service delivery attributes in driving choices in this study. Participants were far less likely to choose a service that had a healthcare worker or peer/lay counselor who was not respectful or understanding (OR 0.186; 95% CI 0.129–0.270 and OR 0.201; 95% CI 0.143–0.285, respectively) compared to a professional healthcare worker who was respectful and understanding. The results of Model 14 (see electronic supplementary material), which separated out the effect of the type of healthcare provider from the attitude of the provider affirmed this finding. There was no significant difference between models delivered by a professional HCW and a peer/lay counselor (OR 0.986; 95% CI 0.891–1.092), but there was a strong and significant difference between a provider who was *not* respectful or understanding compared to provider who *was* respectful and understanding, regardless of any other characteristics of the model (OR 0.441; 95% CI 0.410–0.474).

Participants preferred shorter waiting times over longer waiting times, although time had a comparatively small effect on preferences in relation to the other attributes included in this study. In terms of cost, participants were found to be indifferent between a service costing them US$1 and a free service. However, participants were far less likely to choose a service costing US$3 compared to a service that cost US$1 (OR 0.604; 95% CI 0.497–0.735) or a cost of US$10 compared to a service costing US$1 (OR 0.106; 95% CI 0.068–0.163).

The standard deviation estimates show that there is some significant heterogeneity in preferences between individuals in the sample relating to some of the attribute levels. This is particularly the case for the group vs. individual models and the location of ART delivery (especially home-based vs. clinic-based models). While there is also significant variation in preferences for visit spacing, the magnitude of the standard deviation is relatively small in relation to the mean estimate.

### Interaction Effects and Stratified Analysis

Interaction analysis was used to determine whether there were significant differences in preference structures based on age or sex. The results of the stratified analysis help to understand the preference structures of the different groups. Overall, we found that preference heterogeneity was not very well explained by differences in sex and age. However, Table 5 shows where significant differences in preferences emerged by sex (Model 2) and age (Model 3) in the interaction models.

**Table 5:** Interaction models for gender and age

Attribute (reference level)	Level	Model 2 (sex)		Model 3 (age)		Model 4 (sex and age)		Model 5 (sex and age)		Model 6 (sex and age)		Model 7 (sex and age)	
		Female		18–29 years		Men 30 years and older, and all women		Women 30 and older, and all men		Men 18–29, and all women		Women 18–29, and all men	
		Odds ratio	P-value	Odds ratio	P-value	Odds ratio	P-value	Odds ratio	P-value	Odds ratio	P-value	Odds ratio	P-value
Frequency of visit (every 3 months)	Every 6 months	1.005	0.929	0.055	0.958	1.066	0.130	1.143	0.002	1.059	0.175	1.105	0.018
Individual/group visit (individual)	Group	0.698	< 0.001	0.041	0.708	0.722	0.000	0.786	0.000	0.758	0.000	0.781	0.000
Travel distance (Close to home)	Far from home	0.881	0.155	0.087	0.814	0.881	0.080	0.952	0.500	0.946	0.450	0.938	0.380
	Home based	0.696	0.001	0.074	0.568	0.732	0.000	0.776	0.003	0.720	0.000	0.732	0.000
	Community based	0.790	0.010	0.072	0.661	0.811	0.005	0.812	0.005	0.794	0.002	0.809	0.004
Provider (HCW respectful)	HCW (not respectful)	0.416	< 0.001	0.035	0.320	0.430	0.000	0.423	0.000	0.403	0.000	0.407	0.000
	Peer (respectful)	0.945	0.591	0.102	0.780	0.915	0.297	0.926	0.371	0.958	0.619	0.923	0.348
	Peer (not respectful)	0.470	< 0.001	0.037	0.347	0.447	0.000	0.433	0.000	0.446	0.000	0.410	0.000
Operation times (regular week hours)	Including mornings	0.913	0.337	0.086	0.752	0.905	0.189	0.940	0.413	0.930	0.345	0.901	0.171
	Including evenings	0.843	0.109	0.087	0.661	0.879	0.132	0.947	0.528	0.870	0.109	0.857	0.074
	Including weekends	0.960	0.651	0.079	0.715	0.976	0.741	1.016	0.832	0.945	0.449	0.904	0.176
Waiting time (1 h)	2 h	0.906	0.285	0.078	0.712	0.893	0.131	0.843	0.022	0.861	0.046	0.860	0.041
	4 h	0.899	0.311	0.085	0.650	0.863	0.085	0.817	0.019	0.842	0.046	0.798	0.009
	30 min	1.074	0.438	0.097	0.885	1.129	0.104	1.087	0.264	1.051	0.510	1.119	0.132
Cost (one dollar)	Three dollars	0.795	0.009	0.075	0.706	0.749	0.000	0.731	0.000	0.800	0.002	0.775	0.000
	Ten dollars	0.322	< 0.001	0.041	0.315	0.319	0.000	0.288	0.000	0.326	0.000	0.361	0.000
	Free	1.092	0.313	0.099	0.937	1.041	0.573	0.915	0.208	1.030	0.677	1.052	0.481

		Male		30 years or older		Men 18–29		Women 18–29		Men 30 and older		Women 30 and older	
Frequency of visit (every 3 months)	Every 6 months	1.182	0.022	0.078	0.920	1.106	0.236	0.836	0.036	1.129	0.150	0.962	0.651
Individual/group visit (individual)	Group	1.187	0.019	0.069	0.813	1.237	0.012	0.874	0.116	1.012	0.884	0.901	0.221
Travel distance (Close to home)	Far from home	1.108	0.420	0.116	0.716	1.239	0.150	0.906	0.495	0.924	0.591	0.964	0.805
	Home based	1.120	0.447	0.166	0.836	1.038	0.831	0.818	0.244	1.112	0.533	1.037	0.833
	Community based	1.028	0.829	0.133	0.805	0.969	0.830	0.972	0.853	1.051	0.734	0.968	0.826
Provider (HCW respectful)	HCW (not respectful)	0.990	0.941	0.152	0.910	0.865	0.333	0.900	0.492	1.123	0.435	1.091	0.566
	Peer (respectful)	0.963	0.801	0.140	0.703	1.066	0.716	1.001	0.996	0.892	0.504	1.040	0.819
	Peer (not respectful)	0.839	0.171	0.140	0.850	0.869	0.345	0.996	0.98	0.892	0.444	1.237	0.154
Operation times (regular week hours)	Including mornings	1.010	0.939	0.136	0.792	1.063	0.692	0.900	0.503	0.954	0.755	1.088	0.582
	Including evenings	1.095	0.545	0.177	0.880	1.039	0.828	0.758	0.112	1.084	0.639	1.150	0.426
	Including weekends	0.993	0.956	0.162	0.969	0.939	0.682	0.786	0.107	1.051	0.737	1.274	0.110
Waiting time (1 h)	2 h	0.912	0.475	0.133	0.796	0.868	0.345	1.106	0.503	1.014	0.926	1.029	0.852
	4 h	0.847	0.267	0.161	0.803	0.845	0.335	1.070	0.695	0.950	0.763	1.173	0.359
	30 min	1.040	0.763	0.138	0.827	0.888	0.429	1.031	0.838	1.191	0.241	0.917	0.566
Cost (one dollar)	Three dollars	0.918	0.495	0.103	0.648	1.073	0.628	0.628	0.205	0.833	0.205	0.935	0.639
	Ten dollars	0.997	0.987	0.104	0.516	1.022	0.903	0.903	0.009	0.957	0.797	0.629	0.009
	Free	0.849	0.185	0.101	0.645	0.878	0.367	0.367	0.005	0.920	0.557	0.835	0.200

In the sex interaction model (Model 2) and stratified analysis (Model 8), we found women indifferent between alternative collection schedule options, while men had a significant preference for models containing six-monthly collection options (OR 1.182; 95% CI 1.024–1.364) over three-month collection options compared to women. Men were also found to be more likely than women to choose a group model over an individual model (OR 1.187; 95% CI 1.029–1.370), although the stratified analysis (see Model 8 in the electronic supplementary material) revealed that both men and women preferred group models less than individual models of ART delivery (OR 0.828; 95% CI 0.079–0.088 and OR 0.698; 95% CI 0.630–0.773, respectively).

The age interaction (Model 3) and stratified analysis (Model 9) indicated that older participants in our sample were less willing to pay a fee of US$10 than the younger group (OR = 0.693; 95% CI 0.516–0.930) compared to a fee of US$1. However, the stratified analysis (see Model 9 in the electronic supplementary material) revealed that for both older and younger participants, paying US$10 had the greatest negative effect (OR = 0.269; 95% CI 0.218–0.331 and OR 0.388; 95% CI 0.315–0.478, respectively) compared to a fee of US$1.

**Fig. 3:**
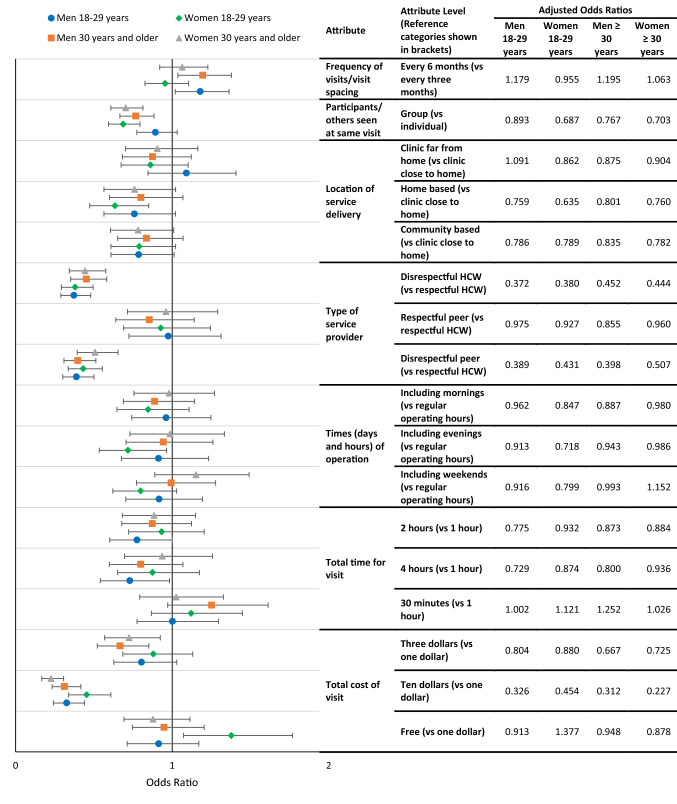
Models 10–13 main effects stratified by age and sex—odds ratios and 95% confidence intervals

Interaction models (Models 4–7) deepened the analysis of preference heterogeneity by examining interactions for sex and age simultaneously (see Table 5). Stratified analysis on both age and sex simultaneously (Models 10–13) revealed that young men were the only sub-set of participants who were indifferent between individual and group models of ART delivery, with all other groups preferring individual over group models. Figure 3 illustrates the results of the main effects analysis stratified by age and sex (Models 10–13). The results of the stratified analysis revealed that location alternatives did not have a significant effect on preferences, with the exception of younger women, who preferred clinic-based collections over home-based delivery (OR = 0.635; 0.473–0.850). Finally, younger women were slightly more willing than other participants to pay US$10 compared to US$1 for services (OR 1.574; 95% CI 1.120–2.210), although overall this still had a highly significant negative effect on preferences in the stratified analysis; for older women, a service that cost US$10 had a significantly stronger negative effect on preferences compared with the other groups (OR 0.629; 95% CI 0.445–0.889). Younger women were the only group for whom a free service was significantly preferable over a cost of US$1 (OR 1.505; 95% CI 1.131–2.003).

## Discussion

Identifying those DART model characteristics which drive patient utilization choices will enable the Zimbabwe MOHCC to prioritize and allocate limited resources more efficiently. The Operational and Service Delivery Manual for the Prevention, Care and Treatment of HIV in Zimbabwe outlines five different ART refill models for stable patients [2]. However, while rolling out all five models simultaneously provides patients a range of options, this is unlikely to be an efficient strategy. Additional guidance is needed on which models to consider prioritizing in different settings to improve efficiency and cost-effectiveness. Provision of ART through differentiated service delivery models requires resources, which if not well aligned to patient preferences, could lead to under-utilization, waste and inefficient provision of services.

The results from this study are useful for understanding patients’ preferences in terms of the characteristics of different DART models. Although our analysis found some evidence of preference heterogeneity, the study found that on the whole, there was a significant preference for clinic-based models compared to community- or home-based models—a finding consistent with a recent DCE from Zambia which found that urban populations were more likely to prefer clinic-based models to models offered in the community [24]. This finding was also supported by qualitative findings, published in more detail elsewhere [9]. Many of the participants in this study reported some symptoms related to their HIV status; thus, having ART delivered through a clinic-based model may provide some assurance that they can access other health services if required. It is also possible that social stigma related to HIV makes community- or home-based models less appealing in urban areas, even though they may be more convenient. ART patients living in rural areas, and indeed some patients in particular urban contexts, may have different preferences regarding the location of services, given that clinics are often further from patients’ homes and more difficult to access; this investigation could be included in future studies and might increase decision-makers’ understanding of the preferences of rural populations in Zimbabwe.

Furthermore, participants often opted for individual over group models. To date, CARGs and other group-based models have had lower uptake in Zimbabwe’s cities than alternative DART models, affirming preference patterns of participants in this study. Group-based models are designed to reduce the amount of time HCWs spend with patients, aiming to reduce patient waiting time in clinics as well as alleviating the burden on HCWs. However, in this study, time was not a highly significant factor in improving participants’ preferences for a specific model. While we found a pattern in the data suggesting that shorter times (including registration, waiting time and consultation) were preferable to longer times, these results were not significant, suggesting that participants were willing to trade off spending a longer time at the facility for engaging with a HCW at an individual level. Based on our findings, one way of improving preferences would be to reduce the number of visits for drug refills. While the magnitude of the effect was relatively small, participants had a significant preference for models that had a 6-monthly drug collection schedule compared to a 3-monthly collection schedule. Reducing the frequency of ARV drug pick-ups is in line with findings from Zambia, where ART patients were found to have a strong preference for a 3-month pick-up schedule compared to a monthly pick-up schedule [24].

Whereas individual-based consultations were preferred to group models, the HCW cadre providing services did not significantly affect preferences. Participants placed significant value on providers who were understanding and respectful over all other characteristics of the delivery models. This is not surprising given the overwhelming evidence showing the negative impact that stigma and discrimination can have on people living with HIV, and particularly on adherence to treatment and retention in care [25–29]. These results replicate those found in a DCE conducted among ART patients who were lost to follow-up in Zambia, confirming that the attitude of HCWs was the most important factor in driving preferences for services [30]. Of concern, previous studies have found that HCWs retain negative attitudes toward people living with HIV, citing issues relating to *inter alia*, personal beliefs, fear of contagion, cultural differences, existing stigma around HIV and discrimination toward people living with HIV that is still pervasive in some communities [28, 31, 32]. Given the importance of this characteristic to patients, DART models must ensure they are staffed by HCWs who are respectful and understanding, in an effort to increase patient satisfaction and utilization. Our finding that patients had no preference regarding models using a professional HCW, lay counselor or peer is encouraging for efforts to adopt task shifting as a way of improving health-system efficiency. This is a critical finding given the recognition that implementation of ART programs must simultaneously focus on increasing efficiencies—task shifting could be a good strategy for achieving this by addressing human resource constraints [33]—and ensuring that patients are retained in care and remain adherent to treatment [6].

Cost was a key characteristic of preference structures and should be an important consideration in the provision of services, not only in terms of user fees, but also when thinking about the costs incurred by patients accessing these services. Almost 80% of study participants reported having to pay a fee of between US$1 and US$3 at their most recent health facility visit. Patients incur transportation costs and experience lost earnings when accessing services. While a small cost of US$1 was found to be bearable and not highly significant in driving choice, we found that a cost of three dollars or ten dollars had a highly significant and large effect on participant preferences.

The operating hours of clinics did not emerge as a significant driver of preferences in our study, seemingly contradictory to findings from research in other contexts [34–36]. Our findings do not necessarily negate the importance of ensuring that clinic operating times are responsive to patients’ needs; however, in relation to the other attributes and levels we included in the DCE design, clinic operating times were not as important in driving preferences as some of the other key attributes. Some of the participants in the qualitative component of the study did raise the importance of clinic operating times, and these findings are discussed in more detail elsewhere [9].

The standard deviation estimates from the mixed effects logit model show that there is significant preference heterogeneity in some attributes. This is important because it shows that although in general preference structures suggest that particular types of delivery models will work better to serve the majority of the population, some people still prefer other types of models. The interaction analyses explored the possibility that the heterogeneity in preferences arose from sex or age characteristics of participants.

Overall, the results of this analysis suggest that preference structures were largely homogenous among men and women, and among older and younger patients. Men, however, favored DART models with less frequent medication collection schedules, explaining some of the heterogeneity present in the mixed effects model standard deviation estimates. This could be linked to the reluctance of men in Zimbabwe to engage with the healthcare system in general [37], or more directly as a result of employment conditions, which make frequent clinic visits prohibitive (63.6% of men in our study were currently employed compared to 38.8% of women). Employment status was, however, not specifically evaluated in the DCE analysis. Some of the heterogeneity in preferences for group vs. individual ART delivery models can be explained by sex – men in our sample (and particularly younger men aged 18–29 years) were more likely than women to choose a group model compared to an individual model. However, this variation was found to be relatively small, and overall, men and women’s preferences were aligned in favor of individual models. The stratified models for sex and age simultaneously showed that location was not a significant driver of choice for any of the groups, with the exception of younger women, who preferred clinic-based services over home-based services. It is possible that for the other groups, this finding is the result of a relatively small sample size to be able to detect weak preferences regarding location. However, it is also possible that preference heterogeneity exists, not on sex and gender, but on other individual characteristics that were not explored in more detail in this analysis. Future studies should investigate this further, as well as other potential sources of heterogeneity among stable HIV patients, including preference structures of adolescents and children, who were ineligible for inclusion in this study.

### Limitations

This study was limited to ART patients living in Harare, an urban area in Zimbabwe; thus, the results are not generalizable to patients in rural areas, or necessarily to other urban areas. Further research is needed to understand the preferences of stable ART patients in other contexts. Secondly, we recognize that some patients may be willing to use services even though the delivery model may not well align with their preferences. Conversely, patients may not use services even though they align with their preference structures for reasons not captured in this study. Additionally, this study used a DCE design with no opt-out option, which maximizes the amount of information about trade-offs, but means there is no reliable anchor for baseline demand or willingness to enroll in DART models. The results of this DCE should thus not be used as a predictor for demand, but rather for understanding overall preference structures and willingness to trade off different service delivery model characteristics. Finally, our study focused on participants who were eligible to be enrolled in a DART model but were still accessing ART services through conventional clinic services at the time of enrollment. The preferences presented in this paper are important for understanding potential barriers and facilitators to enrolling in a DART model, but it is possible that these preference structures change once patients have some experience of actually being enrolled in DART. Thus, further research is required to better understand patient experiences with existing DART models.

## Conclusion

Providing patient-centered services is key to ensuring optimizing and thereby maximizing allocative efficiencies. This paper contributes to the available evidence on the types of DART models that service providers can offer to improve supply side efficiencies in a way that meets the needs and aligns with the preferences of stable ART patients. Models such as the Fast Track, which are clinic-based and individual models, align well with patient preferences; however, these could potentially be modified to increase their appeal to urban patients. While there is some preference heterogeneity between individuals, this is not well explained by age or sex characteristics of participants and further analysis is needed to identify specifically which sub-groups of the population of stable ART patients in Zimbabwe may prefer the different DART models currently being offered. In the meantime, facilities can start by focusing on implementing DART models that are best aligned with the preferences of the majority of patients and improving the characteristics of delivery models that matter most—attitudes of providers and cost of accessing services.

The results of this study are important, not only in the context of ART scale-up in urban Zimbabwe but show how the use of DCEs for eliciting information about patients’ willingness to trade off different characteristics of DART models can be used to provide services that best align with the preferences of patients in particular contexts. The use of these preference data to inform the design of patient-centered services is likely not only to improve the experiences of patients, but ensure optimal uptake of DART services and continued adherence to treatment. This can help to improve health outcomes as well as efficiencies within the health care system.

## Electronic supplementary material

Below is the link to the electronic supplementary material.

Supplementary file1 (XLSX 39 kb)

## Footnotes

1 Four separate dummy variables were created so that, for example, the preference of young men could be assessed in relation to the preferences of everyone else in the sample.
